# Oral health awareness: Periodontitis among diabetic patients in Chhattisgarh, India

**DOI:** 10.6026/973206300211304

**Published:** 2025-06-30

**Authors:** Shashank Agrawal, Vineeta Gupta, Manisha Kumari, Deepesh Kumar Gupta, Komal Sihani, Neha Sahu

**Affiliations:** 1 Department of Periodontics, Government Dental College, Raipur, Chhattisgarh, India; 2 Department of Oral and Maxillofacial Prosthodontics and Implantology, Government Dental College, Raipur, Chhattisgarh, India; 3 Department of Periodontics, Triveni Institute of Dental Sciences, Hospital & Research Centre, Bilaspur Chhattisgarh, India ,Chhattisgarh, India

**Keywords:** Awareness, diabetes mellitus, tribal, hyperglycemia, periodontitis

## Abstract

Awareness on diabetes-periodontitis association among 300 patients (150 diabetic, 150 non-diabetic) attending a tertiary care center in Chhattisgarh is of interest. Only 36% of diabetic participants recognized this link, while 76% of all respondents were unaware of the systemic impact of poor oral hygiene. A mere 24.7% of diabetic individuals had ever been referred to a dentist by a physician. Oral hygiene practices and perceived risk awareness were notably low among both groups. These findings underscore the need for inter-disciplinary efforts and targeted awareness programs to address oral-systemic health gaps.

## Background:

Diabetes mellitus is a group of metabolic disorders characterized by chronic hyperglycemia with disturbances of carbohydrates, fat and protein metabolism resulting from defects in insulin secretion, insulin action, or both [1]. According to WHO, Diabetes Mellitus (DM) stands out as one of the most formidable public health challenges in the 21st century. Presently, it impacts over 422 million individuals throughout the world and by 2030; this number is expected to have doubled. Each year, approximately 1.5 million deaths are directly attributable to diabetes [2]. Prolonged elevated blood glucose levels in patients can cause damage to several organs, including the heart, kidneys, eyes and blood vessels, which eventually results in periodontitis [3]. Periodontal diseases are a group of chronic, progressive bacterial infections resulting in inflammation and destruction of tooth supporting tissues [4]. Periodontitis is a prevalent and progressive disease, impacting roughly 10.5% to 12% of the global population [5]. In 2025, the World Health Organization (WHO) estimates that over 1 billion people globally are affected by severe periodontitis. The main risk factors for periodontal disease are poor oral hygiene and tobacco use [2]. Research findings suggest a link between uncontrolled or inadequately managed diabetes and an elevated vulnerability to infections, especially periodontitis, which may also experience heightened severity under such conditions. Individuals of 45 years or older with poorly controlled diabetes (defined by a glycated hemoglobin level exceeding 9%) are 2.9 times more prone to experience severe periodontitis compared to those without diabetes [6, 7]. The association between periodontitis and diabetes is often characterized as 'two ways' or 'bidirectional' [8]. According to a study conducted by Loein 1993, aggressive periodontitis is recognized as the sixth major complication associated with diabetes [9]. Several studies have examined how maintaining and treating type 2 diabetes and periodontal disease had a reciprocal effect on patient populations. According to recent meta-analyses and systematic reviews, treating periodontal disease may contribute to achieving better glucose control in people with type 2 diabetes [10, 11]. The trials indicated an average reduction in HbA1c ranging from 0.36% to 0.65%. While this may seem like a small adjustment, it's important to know that a 1% drop in HbA1c is connected to a significant 30% decrease in diabetes-related micro-vascular problems [12]. Chhattisgarh, characterized by a significant tribal population, faces challenges related to low levels of social, educational and human development. In the study by Kumar *et al.* approximately 49.9% of individuals resorted to using their finger along with Gudakhu for teeth cleaning, while 35.6% opted for a toothbrush. Twigs were employed by 14.5% of the participants [13]. Given the region's underdevelopment and the high prevalence of poor oral hygiene practices, smoking, alcohol consumption and diabetes, there is an elevated risk of periodontitis [14]. Recognizing the interplay between these prevalent diseases is crucial for their successful treatment and prevention. Dentists, general practitioners and patients all play essential roles in understanding the mutual influence of these conditions. While research has predominantly focused on periodontitis due to its well-established connection with various systemic disorders, a comprehensive approach is necessary for addressing the overall health challenges in the region. According to WHO estimates, the incidence of adults with diabetes is projected to increase from 171 million in 2000 to 366 million worldwide by the year 2030 [15]. According to the latest IDF Diabetes Atlas (2025), 11.1% of the adult populations aged 20-79 years- equivalent to 1 in 9 adults are currently living with diabetes, with more than 40% unaware of their condition. Projections by the IDF estimate that by 2050, around 1 in 8 adults, or approximately 853 million people, will be affected by diabetes, marking a 46% increase [16]. Given the limited awareness among the population of Chhattisgarh regarding the interconnection between diabetes and periodontitis and the potential high prevalence, there is a necessity for a relevant study. Therefore, it is of interest to report awareness levels regarding the bidirectional association between diabetes and periodontitis among patients in Chhattisgarh, along with their oral hygiene practices and attitudes.

## Materials and Methods:

A hospital-based cross-sectional study was conducted among 300 patients (150 diabetic and 150 non-diabetic) attending the outpatient department of Medicine at Dr. Bhimrao Ambedkar Memorial Hospital, Raipur, Chhattisgarh, from May to June 2024. Ethical approval was obtained from the Institutional Ethical Committee of Government Dental College, Raipur (Approval No. ECR/05/GDC/CG/2024) and administrative permission was granted by the Dean of Pt. Jawaharlal Nehru Medical College, Raipur. Informed consent was obtained from all participants. Systematic random sampling was used, selecting every 8th to 10th eligible patient per day starting from a random individual. Equal numbers of diabetic and non-diabetic participants were recruited alternately. A pre-validated, closed-ended questionnaire was administered in the local language via face-to-face interviews [17]. Assistance was provided to participants as required. The questionnaire assessed knowledge of the diabetes-periodontitis association, oral hygiene practices and perceived risk factors. Demographic data were also collected.

## Statistical analysis:

Categorical variables were expressed as frequencies and percentages. The Chi-square test was used to compare categorical data. A significance level of α = 0.05 was considered. Data were analyzed using SPSS for Windows (version 16.0; SPSS Inc., Chicago, IL, USA).

## Results and Discussion:

A total of 300 individuals participated in the study, of which 192 were males and 108 were females. The participants were equally divided into two groups-150 individuals with diabetes and 150 non-diabetic individuals. Demographic information and responses to the questionnaire are summarized in Table 1 (see PDF). Among the diabetic patients, only 36% (n = 54) were aware of the relationship between diabetes and periodontal disease, while a majority (64%, n = 96) lacked this awareness. Regard the source of information, 61.3% of those aware reported receiving information from their dentist, 26.7% from general physicians and the remaining 12.0% from other sources including media or friends. When non-diabetic respondents were asked about their perceived risk of developing diabetes, only 11.3% (n = 17) considered themselves at risk, whereas a significant proportion (88.7%, n = 133) did not perceive any risk. Despite the well-established link between oral health and diabetes, only 24.7% (n = 37) of diabetic participants reported being advised by their physician to consult a dentist for oral health assessment. The majority (75.3%, n = 113) did not receive such recommendations, highlighting a gap in inter-professional collaboration. A majority of the respondents (59.3%, n = 178) believed there was a correlation between their brushing habits and the current status of their gums, indicating an overall recognition of the importance of oral hygiene in maintaining periodontal health. However, when it came to specific knowledge about the association between diabetes and gum diseases, 76% (n = 228) of all respondents were unaware of any connection, indicating a significant knowledge gap in the general population. The study also revealed a lack of consensus among respondents regarding the broader link between systemic health and oral hygiene practices. Although some participants acknowledged the relationship, many expressed uncertainty or insufficient knowledge. These findings underscore the need for comprehensive patient education on the bidirectional relationship between diabetes and periodontal disease and the potential protective role of maintaining good oral hygiene. Both type 1 and type 2 diabetes can have serious side effects from persistently low glycemic control, such as diabetic nephropathy, retinopathy, coronary heart disease and strokes [17]. Diabetes also increases the likelihood of developing dental health problems for those who have it [18] and there appears to be a correlation between glycemic control and the severity of periodontal disease [19]. Figure 1 (see PDF) illustrates the respondents' awareness of the correlation between Diabetes and Periodontitis. Findings revealed that less than a quarter of the respondents possessed a strong comprehension of the link between diabetes and periodontitis.

A systematic review by Maia *et al.* (2020) showed that less than half of people with diabetes are aware of their increased risk for periodontal disease, and dentists are often not the main source of information to motivate such patients [20]. Medical healthcare professionals and dentists should provide mutual care and should consider every patient as a shared responsibility. Inflammation of periodontal tissues triggers inflammatory responses that can exacerbate diabetes, deteriorate cardiovascular outcomes and raise death rates [21]. Nevertheless, A meta-analysis of nine controlled studies found that periodontal treatment could lead to a significant 0.79% reduction in HbA1c levels (95% CI: 0.19 to 1.40), suggesting a clinically meaningful impact on glycemic control in diabetic patients [22]. In 2008, the American Diabetes Association classified periodontitis, as a complication of diabetes. This classification suggested that periodontal disease was a direct result of diabetes and its associated effects on the body. However, a recent publication has proposed a different perspective. Instead of categorizing periodontal disease solely as a complication of diabetes, this publication suggests viewing it as comorbidity-a condition that coexists with diabetes and shares common risk factors and pathways [23]. The distinction between complication and comorbidity is significant because it shifts the understanding of the association between diabetes and periodontitis. Rather than viewing periodontal disease as a direct consequence of diabetes, it is seen as a separate condition that often occurs alongside diabetes due to shared underlying factors. Furthermore, the study mentioned in the prompt highlights a concerning lack of awareness among participants regarding the link between diabetes and periodontitis. Approximately 51.3% of the participants were unaware that diabetes can increase the risk of developing periodontal disease (Table 2 - see PDF). This aligns with findings from previous research studies that have also observed low levels of awareness among patients regarding oral health issues associated with diabetes. One of the surveys on knowledge and awareness about diabetes and periodontal health among Jordanians conducted that 52% of diabetic patients were not aware of the link between diabetes and periodontal health [24]. Further, 62% did not know that treatment of periodontal diseases might help in controlling diabetes mellitus [24]. This lack of awareness underscores the importance of education and outreach efforts aimed at informing individuals with diabetes about the potential risks and implications of periodontal disease. By increasing awareness and understanding of the relationship between diabetes and periodontitis, healthcare providers can empower patients to take proactive steps in managing their oral health and overall well-being. The findings of the current study reveal 57.3% of the participants, lacked awareness of the connection between inadequate oral health and the heightened risk of developing periodontitis. This observation is consistent with a study conducted by Borgnakke and colleagues, which found that individuals with type 2 diabetes or pre-diabetes or no known diabetes, who exhibit poorer periodontal health, also tend to have poorer glycemic control compared to those with better periodontal health [25]. Moreover, a recent meta-analysis that included data from 20 studies, comprising 1 cohort study, 14 case-control studies and 5 cross-sectional studies further supports the correlation between oral health practices and the risk of diabetes. The meta-analysis revealed that a lower frequency of tooth brushing was connected with an increased risk of developing diabetes, with an odds ratio of 1.32 and a 95% confidence interval ranging from 1.19 to 1.47 [26]. In the present study, a significant portion of subjects (57.3%) expressed uncertainty or admitted to lacking knowledge regarding the correlation between poor oral health and the risk of diabetes (Table 4 - see PDF). These findings underscore the significance of oral health in the prevention and management of diabetes. Poor oral hygiene practices and untreated oral health issues can contribute to the development and progression of periodontitis, which in turn may exacerbate glycemic control in individuals with diabetes. Among diabetic individuals (Q6 - Yes), 45.3% believed that being diabetic increases the risk of developing gum-related problems, while 26.7% responded "No" and 28.0% indicated they did not know. Conversely, among non-diabetic individuals (Q6 - No), only 34.7% answered "Yes" to this question, while 6.7% responded "No" and a significant 58.7% were unaware of any association. Notably, the majority of the participants who were aware of the diabetes-periodontal disease link were diabetic (56.7%), while those unaware (*i.e.*, answered "Don't know") were predominantly non-diabetic (67.7%).The Chi-square test revealed a statistically significant association between diabetic status and awareness about diabetes-related gum problems (χ^2^ = 18.205, df = 2, p = 0.001). This indicates that being diabetic is significantly related to higher awareness of the oral health complications associated with diabetes. Additionally, Table 4 (see PDF) highlights a lack of consensus among respondents regarding the potential protective role of good oral hygiene maintenance against diabetes. While a minority (16.0%) believes in its possible protective effect, larger portions (32.7%) do not share this belief. As per Table 3 (see PDF) among diabetic participants, only 36.0% (54) were aware of the connection between diabetes and gum disease, while 64.0% (96) were not. In contrast, among non-diabetic participants, only 9.3% (14) were aware and90.7% (136) were not. This shows that diabetic individuals had significantly higher awareness compared to non-diabetic individuals (36.0% vs 9.3%). However, a large proportion of both groups lacked awareness. The association was found to be statistically significant (Chi-square = 15.213, df = 1, p = 0.001), indicating that diabetic status influences awareness of the relationship between diabetes and gum health. Despite recommendations from organizations like the American Diabetes Association for regular dental check-ups, many diabetes patients do not receive adequate periodontal care, resulting in suboptimal treatment outcomes. The American Diabetes Association advises that patients with diabetes should maintain good oral hygiene and have dental check-ups every six months. Correlating with this in the present study a significant number of respondents (51.3%) expressed uncertainty or lack of knowledge on the protective role of good oral hygiene maintenance against diabetes. Therefore, promoting good oral hygiene habits, including regular tooth brushing and dental check-ups, is crucial for both oral and overall health, particularly in individuals at risk of or living with diabetes. This is particularly important in regions like Chhattisgarh, where there is a large population of tribal people and lower levels of education. Because oral health education and dental care may be harder to come by in these places, prevention is even more important.

In the current study, efforts have been made to gauge the understanding of the well-established reciprocal association between diabetes and periodontitis through a questionnaire-based investigation. Regardless of the importance of oral hygiene studies conducted globally, including those referenced as [27, 28], indicate that people with diabetes frequently have insufficient understanding about the oral and periodontal health complications associated with diabetes. Additionally, they frequently exhibit inadequate oral self-care practices. Several factors might explain this limited awareness. Restricted access to oral healthcare services, especially in rural and tribal areas, is a significant barrier. Many individuals in such regions do not undergo routine dental check-ups unless there is a pressing issue. Furthermore, language barriers and low literacy levels hinder the effectiveness of health communication campaigns. As observed in this study, only 61.3% of participants had ever undergone a blood test to check their glucose levels, reflecting not just limited access, but also a lack of proactive health-seeking behaviour. An important observation from the study is that while 34.7% reported a family history of diabetes, very few had received any information from healthcare providers about the implications of diabetes on oral health. This emphasizes the pivotal role of general physicians and primary care providers in bridging this knowledge gap. Since diabetic patients typically consult general practitioners more frequently than dentists, incorporating oral health education into general medical consultations could substantially improve awareness. Cultural beliefs and local myths prevalent in tribal and underserved communities may also contribute to this gap. In many areas, dental problems are considered minor or unrelated to systemic health. Some individuals may believe that oral diseases are purely age-related or due to dietary habits alone and not linked to chronic conditions like diabetes. Superstitions and mistrust towards modern medical practices further reduce the chances of individuals seeking preventive care or listening to professional advice. There is still a sizable research vacuum concerning the awareness of oral health among people with diabetes, despite the mounting data linking diabetes with problems with oral hygiene. This highlights the need for targeted interventions and educational programs aimed at improving oral health literacy, especially in underserved populations and regions with limited access to healthcare resources. Hence by addressing these knowledge gaps and promoting better oral self-care practices, it is possible to mitigate the risk of oral health complications in individuals with diabetes and improve their overall health outcomes.

## Limitations:

The limitation of the study was due to the restricted sample size. Furthermore, there was no evaluation of potential correlations between occupation, age and profession that might influence knowledge regarding the association between diabetes and periodontitis. Another limitation of lies in the design of the study; being questionnaire-based, it did not involve a clinical assessment of the participants' oral health status or an examination of their dental records.

[1] Ethical approval: Approved by the Institutional Ethical Committee of Government Dental College, Raipur.

IEC No.: ECR/05/GDC/CG/2024

[2] CTRI No.: CTRI/2024/11/076808

## Source of Support and Funding:

Self-Funded

## Conclusion:

Poor awareness of the bidirectional relationship between diabetes and periodontitis, especially among diabetic individuals is a concern. Few participants recognized the systemic implications of oral hygiene or received referrals from physicians for dental evaluation. Hence, the need for interdisciplinary awareness programs to bridge this critical knowledge gap in underserved populations is important.
